# Supplementary material for: Cryo-EM structure and polymorphic maturation of a viral transduction enhancing amyloid fibril
Source: Nat Commun. 2023 Jul 18;14:4293. doi: 10.1038/s41467-023-40042-1 (PMC10354054; doi:10.1038/s41467-023-40042-1)
Supplement: Supplementary file 1 — Supplementary Information [file 41467_2023_40042_MOESM1_ESM.pdf]

Supplementary Information

Cryo-EM structure and polymorphic maturation of a viral transduction enhancing amyloid fibril

Thomas Heerde[a]*, Desiree Schütz[b], Yu-Jie Lin[a], Jan Münch[b],

Matthias Schmidt[a], Marcus Fändrich[a]

Affiliations

[a] Institute of Protein Biochemistry, Ulm University, 89081 Ulm, Germany

[b] Institute of Molecular Virology, Ulm University Medical Center, 89081 Ulm, Germany

Supplementary Methods

Effect of PNF-18 on retroviral infection

The reporter cell line TZM-bl was obtained through the NIH AIDS Reagent Program (Catalogue Number 8129) and cultured in cell culture medium (DMEM medium supplemented with 120 µg/mL penicillin, 120 µg/mL streptomycin, 350 µg/mL glutamine, and 10% inactivated FCS). This cell line is stably transfected with an LTR-lacZ cassette and expresses CD4, CXCR4, and CCR5. Upon HIV-1 infection, the viral protein Tat is expressed, which activates the LTR promotor resulting in the generation of β-galactosidase. To assess the fibril-mediated enhancement of HIV-1 infection, 10^4 TZM-bl cells were seeded in 96 well flat-bottom plates (Sarstedt) the day before infection. Fibrils were preincubated with diluted HIV-1 virus containing ~0.1 ng p24 antigen per mL. After 10 min, 20 µL of these mixtures were added to 180 µl TZM-bl cells, and transduction rates were determined 3 days post-infection by measuring β-galactosidase activities in cellular lysates using the Tropix GalScreen kit (Applied Biosystems, Life Technologies, Frederick, MD) and the Orion microplate luminometer (Berthold). All values give the infection fold increased rates relative to the control, virus only. They are based on the reporter gene activities (RLU/s) derived from triplicate infections minus background activities derived from uninfected cells.

Supplementary Figures

Supplementary Figure 1

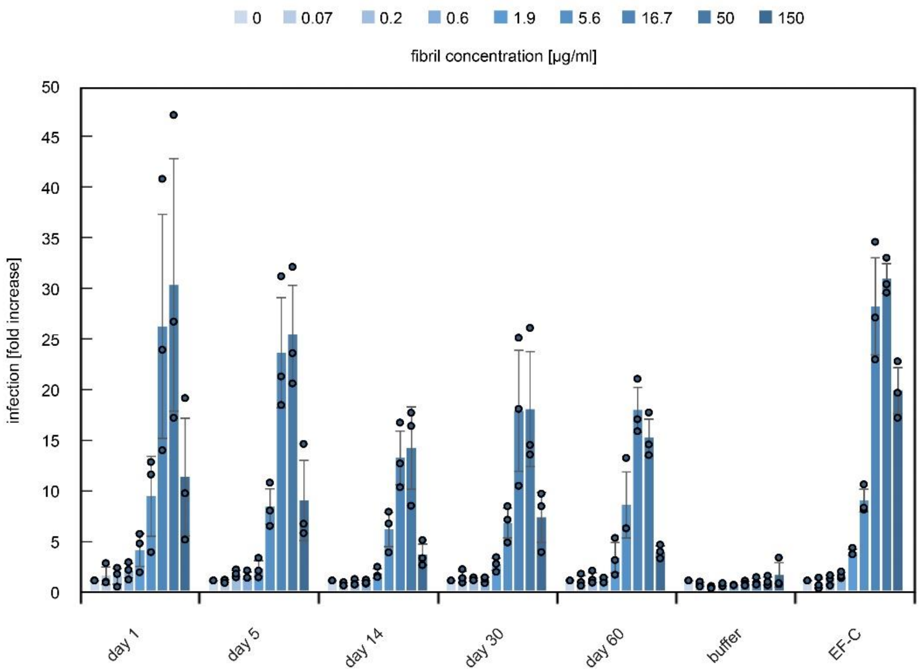

Supplementary Figure 1.

Dependence of the viral infection enhancement on the age of the fibril sample.

HIV-1 particles (0.1 ng p24 antigen/ml) were treated with different concentrations of fibrils (0 to 150 µg/mL) derived from different incubation timepoints (1 to 60 days). Additionally, the parental peptide EF-C was tested as a reference. These mixtures were used to infect TZM-bl cells. The bars give the infection fold increased rates relative to the control, virus only. The black dots represent the single measurements (n=3), the error bars give the standard deviation of the mean fold increase.

Supplementary Figure 2

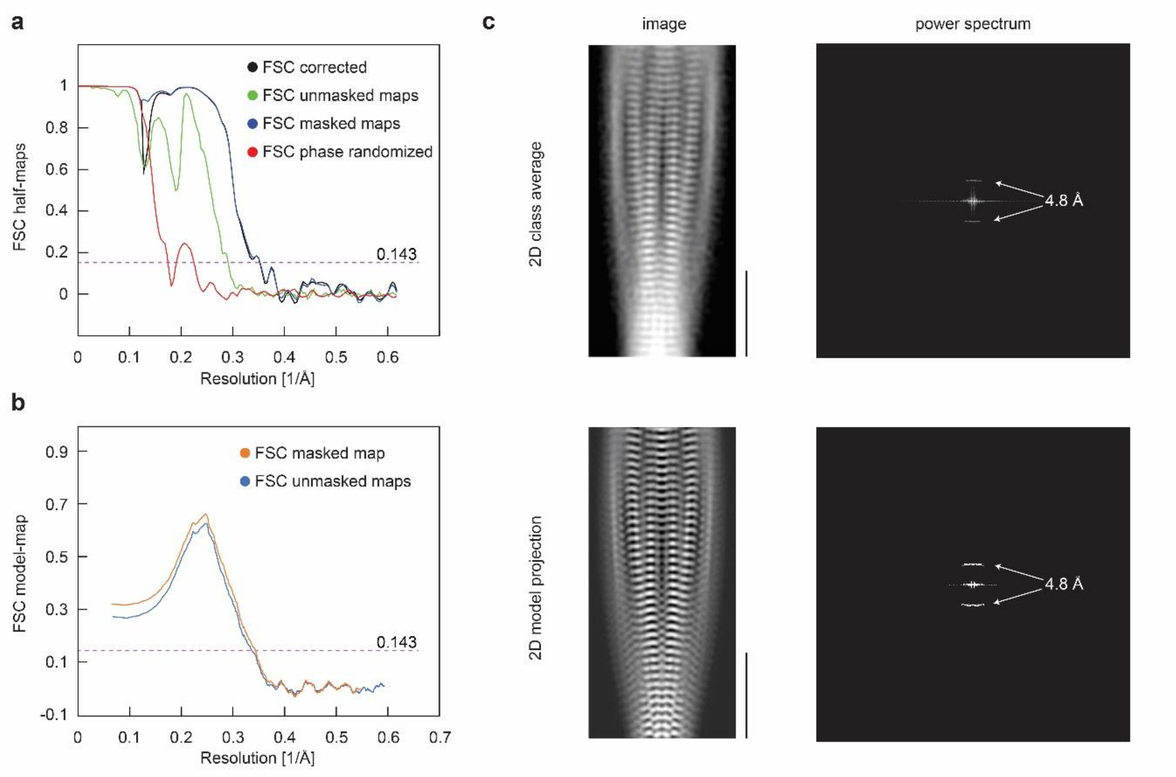

Supplementary Figure 2.

3D map of Morphology II.

(a) FSC of the two half maps of the reconstruction. Black: FSC corrected; green: FSC unmasked maps; blue: FSC masked maps; red: corrected FSC phase randomized masked maps. (b) Model-map FSC. Blue: FSC unmasked maps; orange: FSC masked map. (c) Power spectrum and 2D class average of a consistent part of the fibril (left) and model density projection and power spectrum of the same fibril region (right). Scalebars: 50 Å.

Supplementary Figure 3

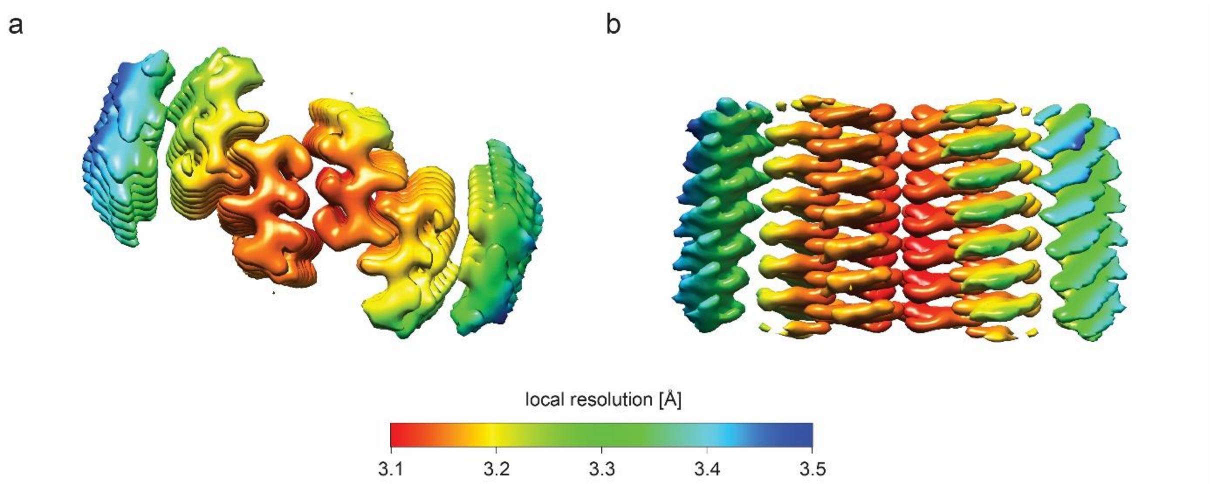

Supplementary Figure 3.

Local resolution of the reconstructed 3D map of Morphology II.

(a) Cross-sectional view of the local resolution of the reconstructed 3D map of Morphology II.

(b) Side view of the local resolution of the reconstructed 3D map of Morphology II. The local resolution was calculated by using relion based on the unmasked half-maps.

Supplementary Figure 4

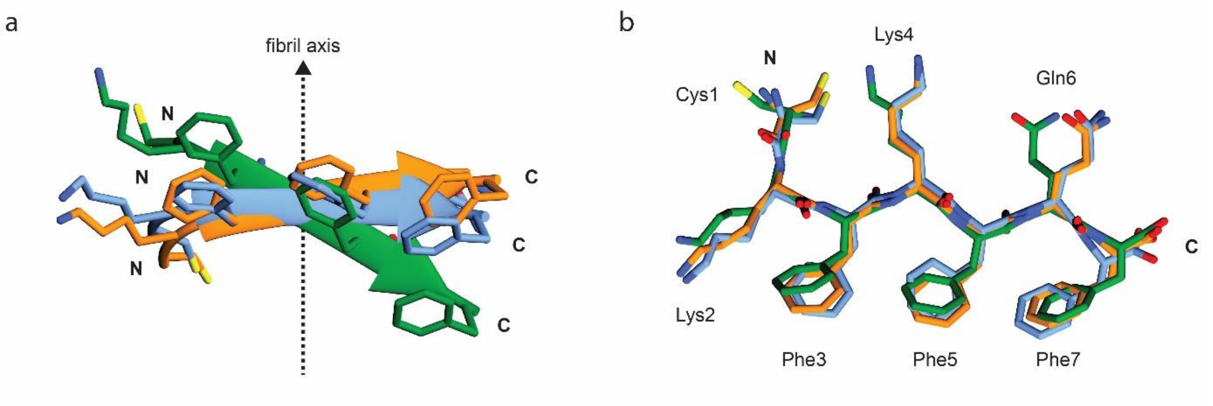

Supplementary Figure 4

Orientation of the peptides of the PNF-18 fibril.

(a) Superimposition of the peptides that form the central protofilament (light blue) and the peripheral protofilament (orange and green) of Morphology II showing their tilt towards the fibril axis. (b) Superimposition of the peptides of Morphology II showing the fluctuation of the sidechains.

Supplementary Figure 5

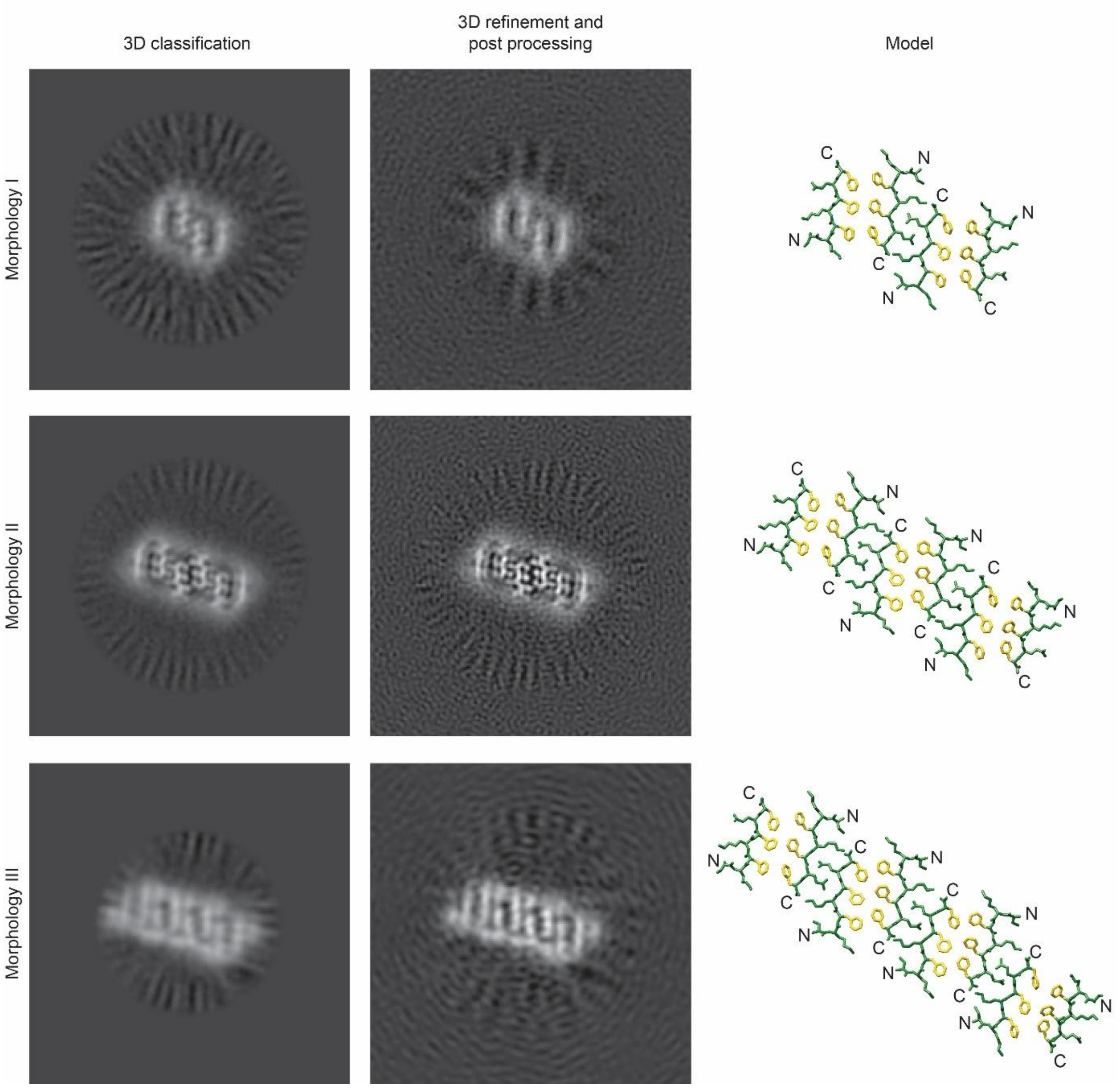

Supplementary Figure 5.

3D reconstruction of Morphology I to III of the PNF-18 fibrils.

Overview of the reconstruction of Morphology I to III, showing the best achieved 3D class (left column) and post processed 3D refinement (middle column). The right column shows the predicted model of Morphology I (top right) and the model of Morphology II (middle right), where the hydrophilic sides are coloured in green and the hydrophobic in yellow.

Supplementary Tables

Supplementary Table 1

**Microscope**	Titan Krios (Thermo Fisher Scientific)
**Camera**	K2 Summit (Gatan)
**Acceleration voltage (kV)**	300
**Magnification**	x 130,000
**Defocus range (μm)**	-0.8 to -2.0
**Dose rate (e^-/Å^2/s)**	5.6
**Number of movie frames**	40
**Exposure time (s)**	8
**Total electron dose (e^-/Å^2)**	45
**Pixel size (Å)**	0.81
**Gatan imaging filter**	20 eV
**Mode**	Counting mode
**Box size (pixel)**	300
**Inter box distance (pixel)**	6
**Number of extracted segments**	356,134
**Number of segments after 2D classification**	297,007
**Number of segments after 3D classification**	25,550
**Unmasked resolution, 0.143 FSC criterion (Å)**	3.42
**Masked resolution, 0.143 FSC criterion (Å)**	2.86
**Map sharpening B-Factor (Å^2)**	-53,24
**Helical rise (Å)**	4.8
**Helical twist (°)**	-2.61
**Symmetry imposed**	C2

Supplementary Table 1.

Structural statistics of cryo-EM data collection and image processing of Morphology II.

Supplementary Table 2

**Model resolution, 0.143 FSC criterion (Å)**	2.9
**Model composition**	
**Non-hydrogen atoms**	3,224
**Protein residues**	168
**Ligands**	0
**RMSDs**	
**Bond length (Å)**	0.007
**Bond angle (°)**	1.748
**Validation**	
**Molprobity score**	0.5
**Clash score**	0
**Poor rotamers (%)**	0
**Ramachandran plot**	
**Favoured (%)**	100
**Allowed (%)**	0
**Disallowed (%)**	0
**EMRinger score**	
**z score**	9.47
**score**	7.31
**Map CC**	
**CCmask**	0.67

Supplementary Table 2.

Structural statistics of model building and refinement.
