# Supplementary material for: Cryo-EM structure and polymorphic maturation of a viral transduction enhancing amyloid fibril
Source: Nat Commun. 2023 Jul 18;14:4293. doi: 10.1038/s41467-023-40042-1 (PMC10354054; doi:10.1038/s41467-023-40042-1)
Supplement: Supplementary file 3 — Reporting Summary [file 41467_2023_40042_MOESM3_ESM.pdf]

nature portfolio

Corresponding author(s): Thomas Heerde

Last updated by author(s): Jul 4, 2023

Reporting Summary

Nature Portfolio wishes to improve the reproducibility of the work that we publish. This form provides structure for consistency and transparency in reporting. For further information on Nature Portfolio policies, see our Editorial Policies and the Editorial Policy Checklist.

Statistics

For all statistical analyses, confirm that the following items are present in the figure legend, table legend, main text, or Methods section.

n/a	Confirmed	
☐	☒	The exact sample size (*n*) for each experimental group/condition, given as a discrete number and unit of measurement
☐	☒	A statement on whether measurements were taken from distinct samples or whether the same sample was measured repeatedly
☒	☐	The statistical test(s) used AND whether they are one- or two-sided<br>*Only common tests should be described solely by name; describe more complex techniques in the Methods section.*
☒	☐	A description of all covariates tested
☒	☐	A description of any assumptions or corrections, such as tests of normality and adjustment for multiple comparisons
☐	☒	A full description of the statistical parameters including central tendency (e.g. means) or other basic estimates (e.g. regression coefficient) AND variation (e.g. standard deviation) or associated estimates of uncertainty (e.g. confidence intervals)
☒	☐	For null hypothesis testing, the test statistic (e.g. *F*, *t*, *r*) with confidence intervals, effect sizes, degrees of freedom and *P* value noted<br>*Give P values as exact values whenever suitable.*
☒	☐	For Bayesian analysis, information on the choice of priors and Markov chain Monte Carlo settings
☒	☐	For hierarchical and complex designs, identification of the appropriate level for tests and full reporting of outcomes
☒	☐	Estimates of effect sizes (e.g. Cohen's *d*, Pearson's *r*), indicating how they were calculated

Our web collection on statistics for biologists contains articles on many of the points above.

Software and code

Policy information about availability of computer code

Data collection	SerialEM v3.7
Data analysis	Relion v3.1, MotionCor v2.1, Gctf v1.06, Coot v0.8.9, PHENIX (MolProbity) v1.16, IMOD v4.9.0, Fiji (ImageJ) 1.52, UCSF Chimera 1.13.1, Situs (pdbsymm) 3.1

For manuscripts utilizing custom algorithms or software that are central to the research but not yet described in published literature, software must be made available to editors and reviewers. We strongly encourage code deposition in a community repository (e.g. GitHub). See the Nature Portfolio guidelines for submitting code & software for further information.

Data

Policy information about availability of data

All manuscripts must include a data availability statement. This statement should provide the following information, where applicable:

- Accession codes, unique identifiers, or web links for publicly available datasets
- A description of any restrictions on data availability
- For clinical datasets or third party data, please ensure that the statement adheres to our policy

The reconstructed cryo-EM map was deposited in the Electron Microscopy Data Bank with the accession codes EMD-16930 [https://www.ebi.ac.uk/emdb/EMD-16930]. The coordinates of the fitted atomic model were deposited in the Protein Data Bank (PDB) under the accession code PDB 8OKR [https://doi.org/10.2210/pdb8OKR/pdb].

Human research participants

Policy information about studies involving human research participants and Sex and Gender in Research.

Reporting on sex and gender	The research shown did not involve human research participants.
Population characteristics	N/A
Recruitment	N/A
Ethics oversight	N/A

Note that full information on the approval of the study protocol must also be provided in the manuscript.

Field-specific reporting

Please select the one below that is the best fit for your research. If you are not sure, read the appropriate sections before making your selection.

☒ Life sciences ☐ Behavioural & social sciences ☐ Ecological, evolutionary & environmental sciences

For a reference copy of the document with all sections, see nature.com/documents/nr-reporting-summary-flat.pdf

Life sciences study design

All studies must disclose on these points even when the disclosure is negative.

Sample size	Fibrils were obtained by incubating 0.3 mg/mL chemically synthesized PNF-18 peptide in 50 mM HEPES buffer, pH 7.0, for a period of up to 60 days at room temperature. The cryo-EM datasets were recored after 1 and 14 days of incubation. The dataset for 1 day incubation that was used for reconstruction contained 6390 micrographs. The sample size was not predetermined, but equals the maximum number of good images that could be taken from the grid. These data were sufficient to lead to a decent reconstruction.
Data exclusions	Initially 356,134 segments were selected. Based on the iterative algorithm of relion, particles that did not align were sorted out. Out of the initial selected segments, 25,550 segments were used for the final reconstruction.
Replication	Electron microscopy data is based on a single sample. 25,550 segments were used for the reconstruction. The retroviral infection assay shown in Supplementary Figure 1 was replicated successfully in three independent experiments (all measurements are shown in SI Fig.1). The dependence of the fibril width on the incubation time was successfully confirmed by 5 images from different positions on the grid (see source data). The measurement of the cross-over distance and the width were successfully replicated 20 times for each morphology.
Randomization	The data shown represents a single case study, therefore randomization is not relevant to study.
Blinding	The data shown represents a single case study, therefore blinding is not relevant to study.

Reporting for specific materials, systems and methods

We require information from authors about some types of materials, experimental systems and methods used in many studies. Here, indicate whether each material, system or method listed is relevant to your study. If you are not sure if a list item applies to your research, read the appropriate section before selecting a response.

Materials & experimental systems

n/a	Involved in the study
☒	☐ Antibodies
☐	☒ Eukaryotic cell lines
☒	☐ Palaeontology and archaeology
☒	☐ Animals and other organisms
☒	☐ Clinical data
☒	☐ Dual use research of concern

Methods

n/a	Involved in the study
☒	☐ ChIP-seq
☒	☐ Flow cytometry
☒	☐ MRI-based neuroimaging

Eukaryotic cell lines

Policy information about cell lines and Sex and Gender in Research

Cell line source(s)	TZM-bl cells were obtained through the NIH AIDS Reagent Program (Catalogue Number 8129)
Authentication	The cell line was authenticated by the AIDS Reagent Program
Mycoplasma contamination	The cells were not tested for mycoplasma
Commonly misidentified lines (See ICLAC register)	No commonly misidentified cell lines were used in this study
